# 2-{[(*E*)-1,3-Benzodioxol-5-yl]methyl­idene­amino}­benzoic acid

**DOI:** 10.1107/S1600536810038420

**Published:** 2010-09-30

**Authors:** M. Nawaz Tahir, Hazoor Ahmad Shad, Muhammad Naeem Khan, Muhammad Ilyas Tariq

**Affiliations:** a Department of Physics, University of Sargodha, Sargodha, Pakistan; b Govt. M. D. College, Department of Chemistry, Toba Tek Singh, Punjab, Pakistan; c Applied Chemistry Research Center, PCSIR Laboratories Complex, Lahore 54600, Pakistan; d Department of Chemistry, University of Sargodha, Sargodha, Pakistan

## Abstract

In the title compound, C_15_H_11_NO_4_, the dihedral angle between the aromatic rings is 23.8 (2)° and an intra­molecular O—H⋯N hydrogen bond generates an *S*(6) ring. In the crystal, C—H⋯O hydrogen bonds link the mol­ecules into a three-dimensional network.

## Related literature

For a related structure, see: Yang *et al.* (2007 ▶). For graph-set notation, see: Bernstein *et al.* (1995 ▶).
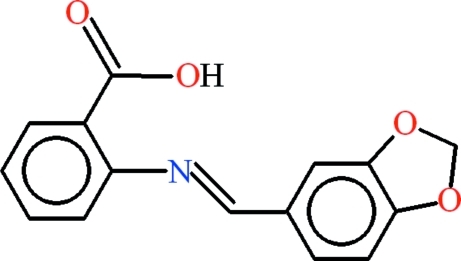


## Experimental

### 

#### Crystal data

C_15_H_11_NO_4_
*M* *_r_* = 269.25
Orthorhombic, 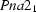

*a* = 22.884 (2) Å
*b* = 3.9402 (4) Å
*c* = 13.5696 (13) Å
*V* = 1223.5 (2) Å^3^
*Z* = 4
Mo *K*α radiation
μ = 0.11 mm^−1^
*T* = 296 K
0.28 × 0.14 × 0.10 mm

#### Data collection

Bruker Kappa APEXII CCD diffractometer
Absorption correction: multi-scan (*SADABS*; Bruker, 2005 ▶) *T*_min_ = 0.980, *T*_max_ = 0.988
28104 measured reflections
1152 independent reflections
925 reflections with *I* > 2σ(*I*)
*R*_int_ = 0.079

#### Refinement

*R*[*F* ^2^ > 2σ(*F* ^2^)] = 0.049
*wR*(*F* ^2^) = 0.122
*S* = 1.12
1152 reflections
184 parameters
1 restraint
H atoms treated by a mixture of independent and constrained refinement
Δρ_max_ = 0.18 e Å^−3^
Δρ_min_ = −0.21 e Å^−3^

### 

Data collection: *APEX2* (Bruker, 2009 ▶); cell refinement: *SAINT* (Bruker, 2009 ▶); data reduction: *SAINT*; program(s) used to solve structure: *SHELXS97* (Sheldrick, 2008 ▶); program(s) used to refine structure: *SHELXL97* (Sheldrick, 2008 ▶); molecular graphics: *ORTEP-3 for Windows* (Farrugia, 1997 ▶) and *PLATON* (Spek, 2009 ▶); software used to prepare material for publication: *WinGX* (Farrugia, 1999 ▶) and *PLATON*.

## Supplementary Material

Crystal structure: contains datablocks global, I. DOI: 10.1107/S1600536810038420/hb5653sup1.cif

Structure factors: contains datablocks I. DOI: 10.1107/S1600536810038420/hb5653Isup2.hkl

Additional supplementary materials:  crystallographic information; 3D view; checkCIF report

## Figures and Tables

**Table 1:** Hydrogen-bond geometry (Å, °)

*D*—H⋯*A*	*D*—H	H⋯*A*	*D*⋯*A*	*D*—H⋯*A*
O1—H1⋯N1	0.83 (7)	1.83 (7)	2.544 (5)	143 (7)
C14—H14⋯O2^i^	0.93	2.42	3.337 (6)	170
C15—H15*A*⋯O2^ii^	0.97	2.60	3.532 (6)	162

Symmetry codes: (i) 
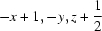
; (ii) 
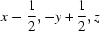
.

**Fig. 1.**
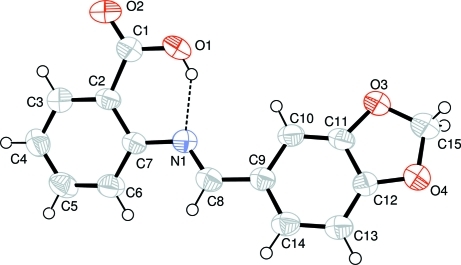
View of (I) with displacement ellipsoids drawn at the 50% probability level. The dotted line represent the intramolecular H-bonding.

**Fig. 2.**
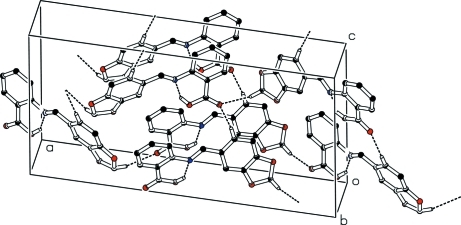
The partial packing of (I), which shows that molecules form polymeric chains.

## supplementary crystallographic information

### Comment

The title compound (I, Fig. 1) is being reported as a part of our on going project related to synthesize various Schiff bases of pipronal and anthranilic acid with different anilines and aldehydes, respectively. The title compound will be utilized for preparing the metal complexes.

The crystal structure of (II) *i.e.*, (*E*)-4-methoxy-*N*-(3,4-methylenedioxybenzylidene)aniline (Yang *et al.*, 2007) has been published which are related to the title compound.

In the title compound, the anthranilic acid moiety A (C1—C7/N1/O1/O2) and pipronal group B (C8—C15/O3/O4) are almost planar with r. m. s. deviations of 0.0105 and 0.0112 Å, respectively. The dihedral angle between A/B is 23.78 (9)°. The intramolecular H-bonding of O—H···N type (Table 1, Fig. 1) complete an S(6) ring motif (Bernstein *et al.*, 1995). The title compound consist of three dimensional zigzag polymeric network (Fig. 2) due to H-bondings of C—H···O type (Table 2). There does not exist any C—H···π interaction.

### Experimental

Equimolar quantities of anthranilic acid and pipronal were refluxed in methanol for 30 min resulting in orange yellow solution. The solution was kept at room temperature which affoarded orange yellow needles of (I) after a week.

### Refinement

In the absence of significant anomalous scattering, all Friedal pairs were merged.

The coordinates of hydroxy H-atom were refined. The carbon H-atoms were positioned geometrically (C–H = 0.93–0.97 Å) and were included in the refinement in the riding model approximation, with *U*_iso_(H) = *xU*_eq_(C, O), where *x* = 1.2 for all H-atoms.

### Crystal data

**Table:**

C_15_H_11_NO_4_	*F*(000) = 560
*M**_r_* = 269.25	*D*_x_ = 1.462 Mg m^−^^3^
Orthorhombic, *P**n**a*2_1_	Mo *K*α radiation, λ = 0.71073 Å
Hall symbol: P 2c -2n	Cell parameters from 925 reflections
*a* = 22.884 (2) Å	θ = 2.3–25.2°
*b* = 3.9402 (4) Å	µ = 0.11 mm^−^^1^
*c* = 13.5696 (13) Å	*T* = 296 K
*V* = 1223.5 (2) Å^3^	Needle, orange yellow
*Z* = 4	0.28 × 0.14 × 0.10 mm

### Data collection

**Table:**

Bruker Kappa APEXII CCD diffractometer	1152 independent reflections
Radiation source: fine-focus sealed tube	925 reflections with *I* > 2σ(*I*)
graphite	*R*_int_ = 0.079
Detector resolution: 8.20 pixels mm^-1^	θ_max_ = 25.2°, θ_min_ = 2.3°
ω scans	*h* = −27→27
Absorption correction: multi-scan (*SADABS*; Bruker, 2005)	*k* = −4→4
*T*_min_ = 0.980, *T*_max_ = 0.988	*l* = −16→16
28104 measured reflections	

### Refinement

**Table:**

Refinement on *F*^2^	Primary atom site location: structure-invariant direct methods
Least-squares matrix: full	Secondary atom site location: difference Fourier map
*R*[*F*^2^ > 2σ(*F*^2^)] = 0.049	Hydrogen site location: inferred from neighbouring sites
*wR*(*F*^2^) = 0.122	H atoms treated by a mixture of independent and constrained refinement
*S* = 1.12	*w* = 1/[σ^2^(*F*_o_^2^) + (0.0704*P*)^2^ + 0.1607*P*] where *P* = (*F*_o_^2^ + 2*F*_c_^2^)/3
1152 reflections	(Δ/σ)_max_ < 0.001
184 parameters	Δρ_max_ = 0.18 e Å^−^^3^
1 restraint	Δρ_min_ = −0.21 e Å^−^^3^

### Special details

**Table:**

Geometry. Bond distances, angles *etc*. have been calculated using the rounded fractional coordinates. All su's are estimated from the variances of the (full) variance-covariance matrix. The cell e.s.d.'s are taken into account in the estimation of distances, angles and torsion angles
Refinement. Refinement of *F*^2^ against ALL reflections. The weighted *R*-factor *wR* and goodness of fit *S* are based on *F*^2^, conventional *R*-factors *R* are based on *F*, with *F* set to zero for negative *F*^2^. The threshold expression of *F*^2^ > σ(*F*^2^) is used only for calculating *R*-factors(gt) *etc*. and is not relevant to the choice of reflections for refinement. *R*-factors based on *F*^2^ are statistically about twice as large as those based on *F*, and *R*- factors based on ALL data will be even larger.

### Fractional atomic coordinates and isotropic or equivalent isotropic displacement parameters (Å^2^)

**Table:**

	*x*	*y*	*z*	*U*_iso_*/*U*_eq_	
O1	0.51893 (16)	0.1333 (11)	0.1270 (2)	0.0783 (14)	
O2	0.60847 (16)	0.3309 (12)	0.1082 (3)	0.0870 (16)	
O3	0.28294 (13)	−0.2221 (9)	0.0939 (2)	0.0644 (11)	
O4	0.22350 (12)	−0.5110 (9)	0.2031 (3)	0.0615 (11)	
N1	0.47183 (15)	0.1225 (8)	0.2968 (3)	0.0477 (11)	
C1	0.5677 (2)	0.2642 (12)	0.1623 (3)	0.0597 (17)	
C2	0.56974 (18)	0.3361 (10)	0.2714 (3)	0.0453 (12)	
C3	0.62059 (19)	0.4809 (11)	0.3079 (4)	0.0557 (16)	
C4	0.6268 (2)	0.5551 (12)	0.4064 (4)	0.0603 (17)	
C5	0.5811 (2)	0.4898 (11)	0.4697 (4)	0.0613 (17)	
C6	0.53019 (19)	0.3494 (11)	0.4360 (3)	0.0550 (16)	
C7	0.52366 (16)	0.2630 (10)	0.3363 (3)	0.0427 (12)	
C8	0.43348 (17)	−0.0263 (9)	0.3505 (3)	0.0487 (12)	
C9	0.37893 (17)	−0.1565 (9)	0.3122 (3)	0.0453 (12)	
C10	0.36230 (17)	−0.1111 (11)	0.2122 (3)	0.0480 (12)	
C11	0.30981 (17)	−0.2372 (11)	0.1848 (3)	0.0463 (12)	
C12	0.27312 (17)	−0.4075 (11)	0.2501 (3)	0.0477 (14)	
C13	0.28753 (18)	−0.4521 (11)	0.3468 (4)	0.0520 (14)	
C14	0.34183 (19)	−0.3234 (11)	0.3770 (3)	0.0520 (14)	
C15	0.2282 (2)	−0.3962 (14)	0.1039 (4)	0.0650 (17)	
H1	0.491 (3)	0.140 (15)	0.166 (5)	0.0937*	
H3	0.65112	0.52880	0.26487	0.0668*	
H4	0.66140	0.64831	0.43000	0.0722*	
H5	0.58489	0.54176	0.53624	0.0734*	
H6	0.49957	0.31078	0.47961	0.0655*	
H8	0.44114	−0.05185	0.41734	0.0582*	
H10	0.38644	0.00051	0.16763	0.0575*	
H13	0.26261	−0.56229	0.39043	0.0623*	
H14	0.35328	−0.35079	0.44231	0.0623*	
H15A	0.19620	−0.24411	0.08841	0.0780*	
H15B	0.22671	−0.58749	0.05902	0.0780*	

### Atomic displacement parameters (Å^2^)

**Table:**

	*U*^11^	*U*^22^	*U*^33^	*U*^12^	*U*^13^	*U*^23^
O1	0.071 (2)	0.130 (3)	0.034 (2)	−0.009 (2)	−0.0057 (16)	−0.008 (2)
O2	0.074 (2)	0.143 (4)	0.044 (2)	−0.012 (2)	0.0141 (19)	−0.003 (2)
O3	0.0587 (18)	0.094 (2)	0.0405 (18)	−0.0102 (18)	−0.0039 (15)	0.0052 (17)
O4	0.0574 (17)	0.080 (2)	0.047 (2)	−0.0098 (16)	−0.0032 (15)	0.0034 (18)
N1	0.0472 (19)	0.055 (2)	0.041 (2)	0.0002 (16)	0.0019 (17)	0.0018 (17)
C1	0.055 (3)	0.079 (3)	0.045 (3)	0.000 (2)	−0.002 (2)	0.004 (2)
C2	0.052 (2)	0.049 (2)	0.035 (2)	0.0056 (19)	0.0013 (18)	0.0029 (18)
C3	0.053 (2)	0.065 (3)	0.049 (3)	−0.002 (2)	0.002 (2)	−0.001 (2)
C4	0.057 (3)	0.068 (3)	0.056 (3)	−0.007 (2)	−0.011 (2)	−0.002 (2)
C5	0.079 (3)	0.062 (3)	0.043 (3)	−0.008 (3)	−0.007 (3)	−0.008 (2)
C6	0.063 (3)	0.059 (2)	0.043 (3)	−0.003 (2)	0.004 (2)	−0.002 (2)
C7	0.048 (2)	0.044 (2)	0.036 (2)	0.0044 (18)	−0.0005 (18)	0.0011 (16)
C8	0.055 (2)	0.049 (2)	0.042 (2)	0.007 (2)	0.000 (2)	0.002 (2)
C9	0.048 (2)	0.047 (2)	0.041 (2)	0.0048 (18)	0.0014 (19)	0.0020 (18)
C10	0.050 (2)	0.054 (2)	0.040 (2)	0.0019 (19)	0.006 (2)	0.0058 (19)
C11	0.052 (2)	0.054 (2)	0.033 (2)	0.009 (2)	0.0016 (19)	−0.0006 (19)
C12	0.045 (2)	0.055 (2)	0.043 (3)	−0.0001 (19)	0.0020 (19)	−0.001 (2)
C13	0.056 (2)	0.055 (2)	0.045 (3)	0.000 (2)	0.006 (2)	0.006 (2)
C14	0.056 (2)	0.054 (2)	0.046 (3)	0.008 (2)	0.006 (2)	0.010 (2)
C15	0.067 (3)	0.082 (3)	0.046 (3)	−0.003 (3)	−0.005 (2)	0.006 (2)

### Geometric parameters (Å, °)

**Table:**

O1—C1	1.320 (6)	C9—C14	1.388 (6)
O2—C1	1.216 (6)	C9—C10	1.421 (6)
O3—C11	1.380 (5)	C10—C11	1.352 (6)
O3—C15	1.435 (6)	C11—C12	1.393 (6)
O4—C12	1.365 (5)	C12—C13	1.364 (7)
O4—C15	1.424 (7)	C13—C14	1.403 (6)
O1—H1	0.83 (7)	C3—H3	0.9300
N1—C7	1.414 (5)	C4—H4	0.9300
N1—C8	1.283 (5)	C5—H5	0.9300
C1—C2	1.508 (6)	C6—H6	0.9300
C2—C7	1.404 (6)	C8—H8	0.9300
C2—C3	1.387 (6)	C10—H10	0.9300
C3—C4	1.376 (8)	C13—H13	0.9300
C4—C5	1.378 (7)	C14—H14	0.9300
C5—C6	1.368 (6)	C15—H15A	0.9700
C6—C7	1.403 (6)	C15—H15B	0.9700
C8—C9	1.446 (5)		
			
C11—O3—C15	106.5 (3)	C11—C12—C13	121.9 (4)
C12—O4—C15	106.5 (3)	O4—C12—C11	110.4 (4)
C1—O1—H1	114 (5)	C12—C13—C14	116.6 (4)
C7—N1—C8	122.5 (4)	C9—C14—C13	121.9 (4)
O1—C1—O2	121.0 (4)	O3—C15—O4	107.9 (4)
O1—C1—C2	117.1 (4)	C2—C3—H3	119.00
O2—C1—C2	121.9 (4)	C4—C3—H3	119.00
C1—C2—C3	117.0 (4)	C3—C4—H4	120.00
C1—C2—C7	123.7 (4)	C5—C4—H4	120.00
C3—C2—C7	119.4 (4)	C4—C5—H5	120.00
C2—C3—C4	121.4 (4)	C6—C5—H5	120.00
C3—C4—C5	119.2 (4)	C5—C6—H6	120.00
C4—C5—C6	120.9 (5)	C7—C6—H6	120.00
C5—C6—C7	120.7 (4)	N1—C8—H8	118.00
N1—C7—C2	118.2 (4)	C9—C8—H8	118.00
N1—C7—C6	123.4 (4)	C9—C10—H10	121.00
C2—C7—C6	118.4 (4)	C11—C10—H10	122.00
N1—C8—C9	123.3 (4)	C12—C13—H13	122.00
C8—C9—C14	117.9 (4)	C14—C13—H13	122.00
C10—C9—C14	120.1 (4)	C9—C14—H14	119.00
C8—C9—C10	122.0 (4)	C13—C14—H14	119.00
C9—C10—C11	117.0 (4)	O3—C15—H15A	110.00
O3—C11—C10	128.8 (4)	O3—C15—H15B	110.00
O3—C11—C12	108.7 (3)	O4—C15—H15A	110.00
C10—C11—C12	122.5 (4)	O4—C15—H15B	110.00
O4—C12—C13	127.7 (4)	H15A—C15—H15B	109.00
			
C15—O3—C11—C10	179.6 (5)	C3—C4—C5—C6	0.7 (7)
C15—O3—C11—C12	−1.2 (5)	C4—C5—C6—C7	1.1 (7)
C11—O3—C15—O4	0.5 (5)	C5—C6—C7—N1	−178.9 (4)
C15—O4—C12—C11	−1.1 (5)	C5—C6—C7—C2	−2.4 (6)
C15—O4—C12—C13	178.3 (5)	N1—C8—C9—C10	−3.7 (6)
C12—O4—C15—O3	0.4 (5)	N1—C8—C9—C14	177.8 (4)
C8—N1—C7—C2	162.9 (4)	C8—C9—C10—C11	−178.7 (4)
C8—N1—C7—C6	−20.6 (6)	C14—C9—C10—C11	−0.2 (6)
C7—N1—C8—C9	176.8 (3)	C8—C9—C14—C13	178.7 (4)
O1—C1—C2—C3	−178.6 (4)	C10—C9—C14—C13	0.2 (6)
O1—C1—C2—C7	1.8 (6)	C9—C10—C11—O3	178.6 (4)
O2—C1—C2—C3	−0.4 (7)	C9—C10—C11—C12	−0.5 (6)
O2—C1—C2—C7	−180.0 (5)	O3—C11—C12—O4	1.5 (5)
C1—C2—C3—C4	−179.8 (4)	O3—C11—C12—C13	−178.0 (4)
C7—C2—C3—C4	−0.1 (6)	C10—C11—C12—O4	−179.3 (4)
C1—C2—C7—N1	−1.8 (6)	C10—C11—C12—C13	1.3 (7)
C1—C2—C7—C6	−178.5 (4)	O4—C12—C13—C14	179.4 (4)
C3—C2—C7—N1	178.6 (4)	C11—C12—C13—C14	−1.2 (6)
C3—C2—C7—C6	1.9 (6)	C12—C13—C14—C9	0.5 (6)
C2—C3—C4—C5	−1.2 (7)		

### Hydrogen-bond geometry (Å, °)

**Table:**

*D*—H···*A*	*D*—H	H···*A*	*D*···*A*	*D*—H···*A*
O1—H1···N1	0.83 (7)	1.83 (7)	2.544 (5)	143 (7)
C14—H14···O2^i^	0.93	2.42	3.337 (6)	170
C15—H15A···O2^ii^	0.97	2.60	3.532 (6)	162

Symmetry codes: (i) −*x*+1, −*y*, *z*+1/2; (ii) *x*−1/2, −*y*+1/2, *z*.
